# A Muscle-Specific Rehabilitation Training Method Based on Muscle Activation and the Optimal Load Orientation Concept

**DOI:** 10.1155/2018/2365983

**Published:** 2018-11-22

**Authors:** Zhibin Song, Chao Nie, Shuyang Li, Paolo Dario, Jian S. Dai

**Affiliations:** ^1^Key Laboratory of Mechanism Theory and Equipment Design of Ministry of Education, Tianjin University, Tianjin 300072, China; ^2^The BioRobotics Institute, Scuola Superiore Sant'Anna, Polo Sant'Anna Valdera, V.le R. Piaggio 34, 56025 Pontedera, Italy; ^3^School of Natural and Mathematical Sciences, King's College London, University of London, Strand London WC2R 2LS, UK

## Abstract

Training based on muscle-oriented repetitive movements has been shown to be beneficial for the improvement of movement abilities in human limbs in relation to fitness, athletic training, and rehabilitation training. In this paper, a muscle-specific rehabilitation training method based on the optimal load orientation concept (OLOC) was proposed for patients whose motor neurons are injured, but whose muscles and tendons are intact, to implement high-efficiency resistance training for the shoulder muscles, which is one of the most complex joints in the human body. A three-dimensional musculoskeletal model of the human shoulder was used to predict muscle forces experienced during shoulder movements, in which muscles that contributed to shoulder motion were divided into 31 muscle bundles, and the Hill model was used to characterize the force-length properties of the muscle. According to the musculoskeletal model, muscle activation was calculated to represent the muscle force. Thus, training based on OLOC was proposed by maximizing the activation of a specific muscle under each posture of the training process. The analysis indicated that the muscle-specific rehabilitation training method based on the OLOC significantly improved the training efficiency for specific muscles. The method could also be used for trajectory planning, load magnitude planning, and evaluation of training effects.

## 1. Introduction

Rehabilitation robots that provide rehabilitation therapy following neurological injuries, such as stroke and spinal cord injury, have received increasing interest [1]. The shoulder complex is one of the most complicated joints in the human body, as it directly affects the performance of the whole upper limb movement, including hand manipulations. After suffering from a neurological disease, the motor function of the human shoulder complex can often face deterioration, and there is an increased risk of spasticity; thus, a proper approach to motor function training therapy is necessary. Previous studies have also indicated that the use of rehabilitation robotics can provide a high-intensity, task-oriented, and highly repetitive treatment in the impaired upper limb, which have all been shown to be beneficial for the restoration of shoulder function [2–8]. Rehabilitation robots can train patients' limb movements in several ways by applying turnable forces to patients. Training with active-resistance mode rehabilitation robots can actively deliver resistance against movements executed by the patient. Accurate rehabilitation for human limb motor function has been widely accepted and has become increasingly popular; accurate rehabilitation requires both a higher level of control and smarter robotic designs as well as increasingly accurate models of the human motor system. However, the rehabilitation efficiency of current methods is still controversial [9–11] because training methods are often based on the clinical experience of the doctor or therapist who chooses from standard menus and options rather than making precise decisions based on the actual situation present in the patients' limb [10, 12]. By contrast, most studies of rehabilitation robotics are focused on rehabilitating a whole limb, so loads often cannot be applied to a specific muscle, which is important in the rehabilitation training process because different muscles often have different levels of damage, stiffness, or deterioration. To apply efficient and targeted training to a specific muscle, Terashima et al. and Itokazu et al. [13–15] proposed the concept of specific-muscle training for the upper limbs using optimization control of patients' neural network and via EMG feedback control methods. The results showed that this training method aimed at specific muscles maximized the training effect in target muscles and simultaneously weakened the effects on other muscles. However, their research was limited to cases of small-scale movements within a single horizontal plane, and the need for real-time EMG signals as an input value has limited this method's practicality.

Compared with most developers of rehabilitation robotics, researchers in biomedical engineering have focused on studying the physiological musculoskeletal model of the human shoulder. For shoulder models, the typical representations of the muscles' lines of action are the line-segment model [11, 16–18] and more complex 3D finite element model [19]. These models were intended to be used for several purposes, such as surgical simulation [17], wheelchair mechanics research [20, 21], neuroprostheses control [22, 23], and other similar applications. Most current studies using a musculoskeletal model focus on joint-contact forces and muscle moment arms. The direct purpose of these studies was to reproduce and simulate the patterns of muscle force generation. However, musculoskeletal models based on the anatomical structure have not been adequately or currently applied to rehabilitation robots [10, 24]. Therefore, in this paper, a three-dimensional mathematical musculoskeletal model [16, 25, 26] of the shoulder complex was used to represent the relationship of muscles and external loads. Based on this model, the static forces that acted on all of the shoulder bones, including the humerus, scapula, and clavicle as well as the muscles connected to these bones, were analysed and calculated. Muscle activation was proposed to measure the force exhibited by a certain muscle undergoing an isometric contraction. The optimal load orientation (OLO) for muscle rehabilitation was then obtained according to the results of the calculated muscle activation. Finally, a specific muscular rehabilitation training approach based on the optimal load orientation concept (OLOC) was proposed.

Rehabilitation robots were controlled to apply forces to each joint axis separately via intelligent application of the exoskeleton mechanism. For a given rehabilitation movement trajectory of the shoulder, the optimal load orientation of a specific muscle of the whole range of motion (optimal load orientation cluster) was determined by calculating an inverse dynamic problem. Then, the shoulder movement trajectory of rehabilitation was designed or evaluated according to the optimal load orientation cluster above. The results showed that training based on the OLOC enhanced the activation of specific muscles and reduced the activation of other muscles and thus enabled efficient training of specific muscles.

This paper also assessed the influence of the magnitude of the external load on the training effects of OLOC training through simulation testing.

## 2. Materials and Methods

When implementing active-resisted rehabilitation training for an impaired shoulder using a robotic device, robots delivered resistance against active movements executed by the shoulder.

During shoulder movement, for a specific muscle, a certain external load with different orientations leads to different muscle forces. Therefore, for a specific muscle, muscle activation can be designed by controlling the orientation of the external load.

### 2.1. Musculoskeletal Model of the Shoulder Complex

**Figure 1:**
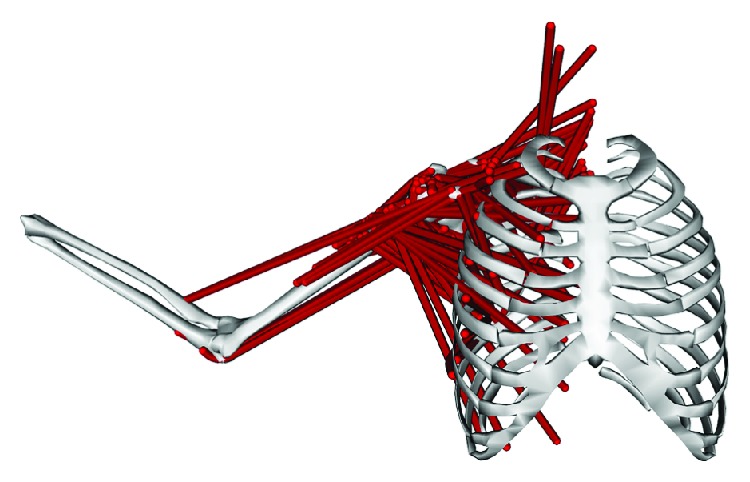
Visual representation of a musculoskeletal model of the shoulder.

A three-dimensional mathematical musculoskeletal shoulder model [16, 25, 26] was used, as shown in Figure 1 [27], to represent the geometric architectural properties of the skeleton and muscles of the shoulder. The geometric parameters of the model were developed using CT images of bones and muscles collected from the Visible Human Project (VHP) database [25].

#### 2.1.1. Skeleton Model

The shoulder skeletal structure consists of the following bones: the thorax, clavicle, scapula, and humerus, and the following joints: the sternoclavicular joint (SC), acromioclavicular joint (AC), glenohumeral joint (GH), and scapulothoracic joint (ST).

**Figure 2:**
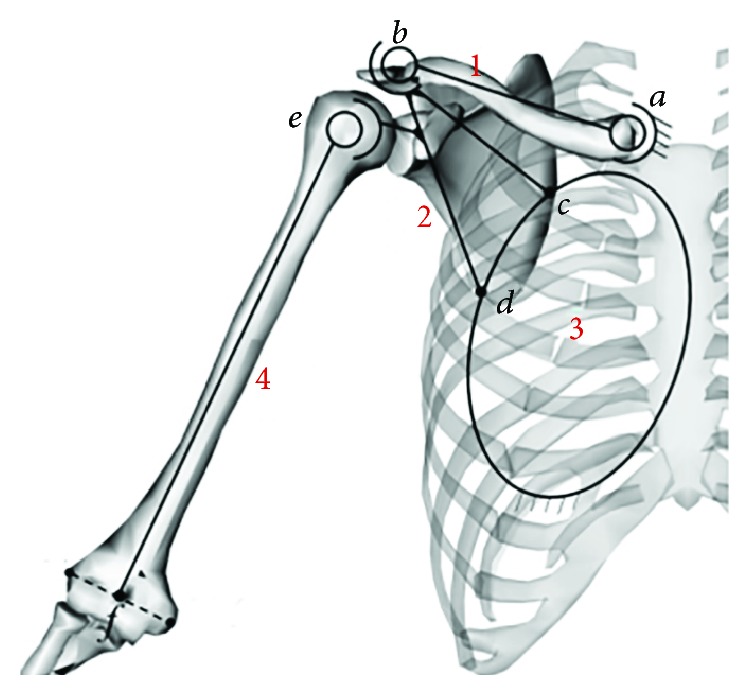
Schematic diagram of the shoulder mechanism.

In this paper, a hybrid mechanism model was used to simulate the structure of the skeletal system of the shoulder, as depicted in Figure 2, where “1” represents the clavicle, “2” represents the scapula, “3” represents the thorax, and “4” represents the humerus; point *a* represents the SC articulation; *b* represents the AC articulation; *c* and *d* represent the upper feature point and lower feature point of the scapula, respectively; and *e* represents the midpoint of the EL (lateral epicondyle) and EM (medial epicondyle) in the humerus.

Considering that translations are negligible compared with rotations, the SC, AC, and GH joints were assumed to be ball-and-socket joints. As an exception, the ST joint was considered to be a joint that allowed the scapula translation and rotator movement with respect to the thorax due to the compliance of the surrounding muscles. The thorax was represented as an ellipsoid, as shown in Figure 2.

To describe and analyse the skeleton model above, every bone is fixed to a coordinate system, as shown in Table 1. The ISB standard recommended by Wu et al. [28] is widely used in the field of biomedical engineering to describe the movements of bones and joints in the human upper limb. The relationship between each coordinate system described in Table 1 and its corresponding coordinate system recommended in the ISB standard can be described by a rotation transformation matrix.

#### 2.1.2. Muscle-Driven Model and Muscle Activation

The eighteen muscle groups associated with shoulder motion were divided into 31 muscle bundles according to the results of anatomical measurements of shoulder muscles performed by Garner and Pandy [26], and these muscle bundles were numbered from M1 to M31, as shown in Table 2.

To calculate the muscle force, each muscle bundle was modelled as a 3-element Hill-type model that was widely applied in muscle-driven simulations [29–32]. Four parameters were used to represent each muscle's force-generating properties, including the tendon slack length (*L*_*s*_^*T*^), pennation angle (*β*), optimal muscle-fibre length (*L*_*o*_^*M*^), and peak isometric muscle force (*F*_*o*_^*M*^). The values of the parameters of each muscle bundle are shown in Table 2 and were determined from the reports by Garner and Pandy [33] and Yanagawa et al. [34].

According to the Hill model, the actual muscle-fibre length (*L*^*M*^) and isometric muscle force (*F*^*M*^) can be calculated using the data presented in Table 1, and the total muscle length *L* of the muscle bundle was determined in the musculoskeletal model by the obstacle-set method proposed by Garner and Pandy [26].

During shoulder movements, the muscle length changes with bone movements. When the shoulder is in a certain position and posture, the muscles can exhibit contractions that are considered isometric, so the actual muscle force *F* can change while the muscle length remains unchanged. Muscle activation *a* is defined by the equation below to describe the force state of a muscle during an isometric contraction:

(1) $$\begin{array}{c} a=\frac{F}{F^{M}},\;0\leq a\leq 1. \end{array}$$

When the actual force is maximum, *F* = *F*^*M*^ and *a* = 1; when the actual force is minimum, *F* = 0 and *a* = 0.

#### 2.1.3. Musculoskeletal Model

Inside the human body, muscles are often observed to be bar-like fibres and are connected to the bones via tendons. Muscles will always bypass some bones, joints, and surrounding tissue that forms the muscle path that passes the origin, via point, obstacle, and point of insertion [26]. Based on the skeletal and muscle models depicted above, the muscle path was determined using the obstacle-set method proposed by Garner and Pandy [26]. Here, the data used in the obstacle-set method, such as the position of the feature points (origin point or insertion point) and type and size of the obstacles, were determined from the results reported by Garner and Pandy [16, 26], and all data were transformed into the coordinate system that is described in Table 1. Once the path of a muscle bundle was determined, its total muscle length was determined. The actual muscle-fibre length (*L*^*M*^) and isometric muscle force (*F*^*M*^) were then calculated.

### 2.2. Static Analysis and Prediction of Muscle Activation

Muscle activation *a* was used to describe the force condition of the muscle bundles. In this paper, inertial forces were ignored because the motion of the shoulder is slow; thus, the shoulder could be considered to be in a situation of static balance. Some multisolution static equilibrium problems were solved to calculate the muscle forces for each prescribed posture of the shoulder under a certain external load. Any shoulder bone is simultaneously affected by gravity, joint force, joint torque, and muscle torque. The exerted torque of a muscle on any shoulder bone could be determined by static analysis of the bone. Once the muscle torques were obtained, the static balance equations describing the muscles were established and the activation of each muscle bundle was calculated by solving the equations.

#### 2.2.1. Static Analysis of Bones

The gravity of the bones and muscles of the upper limbs was considered to be one force, while the mass and centroid position of the upper limbs were determined based on the results of anthropometrical data from the study by Shan and Bohn [35]. When the shoulder is in a certain posture, the static balance equations describing the humerus, scapula, and clavicle can be established, and then, the muscle input torques that are needed to drive each bone can be calculated by solving the equations.

**Figure 3:**
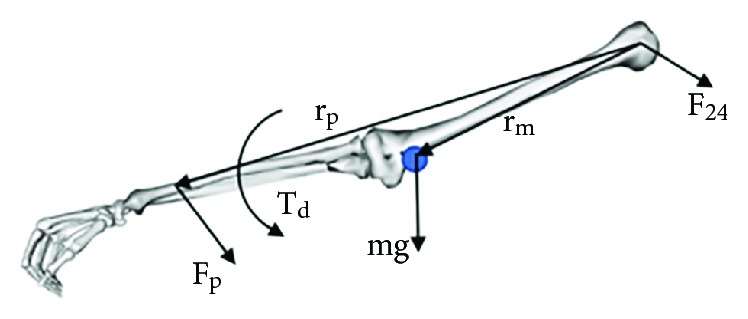
Free body diagrams of the upper limb.

The humerus was analysed as an example. To indicate the force between two bones, *F*_*ij*_ was used to represent the force that is applied to bone *j* by bone *i* (Figure 2). The free body diagram of the upper limb bones is shown in Figure 3, where mg is the gravitational force on the upper limb, *F*_*p*_ is the external load, *F*_24_ is the force applied to the humerus by the scapula, *r*_*m*_ is the position vector of the upper limb centroid, *r*_*p*_ is the positioning vector for the point where the external force is applied, and *T*_*d*_ is the input torque applied to the upper limb produced by the muscles. Since the GH joint was assumed to be a ball-and-socket joint, the joint torque applied to the humerus by the scapula through the GH joint was described as zero.

The value of each vector was determined in the global coordinate *S*_0_. The joint force *F*_24_ and the input torque *T*_*d*_ were calculated by solving the static equilibrium equations

(2) $$\begin{array}{c} F_{24}+mg+F_{p}=0, \end{array}$$

(3) $$\begin{array}{c} T_{d}+r_{m}\times mg+r_{p}\times F_{p}=0. \end{array}$$

**Figure 4:**
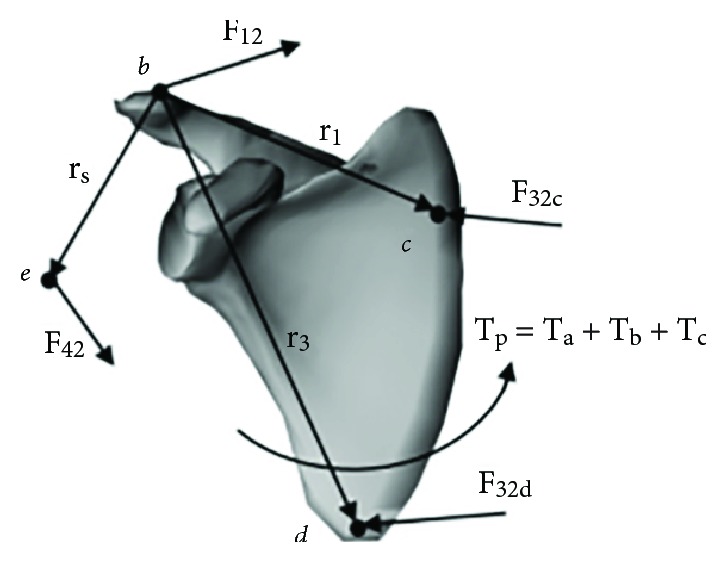
Free body diagrams of the scapula.

**Figure 5:**
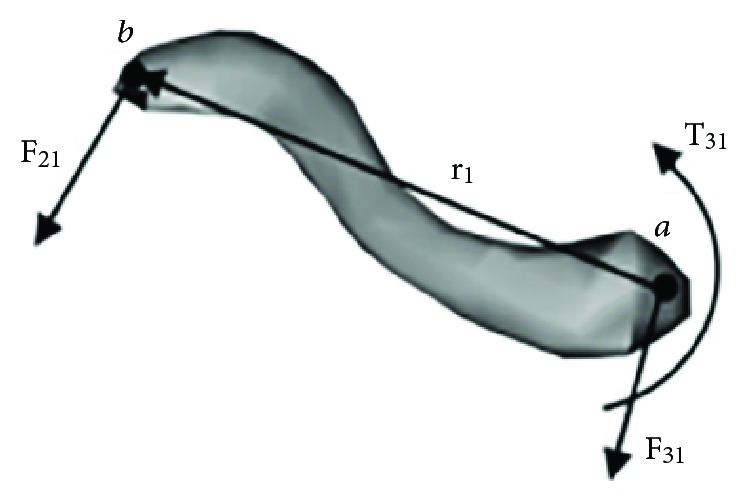
Free body diagrams of the clavicle.

Similarly, static analyses of the scapula and the clavicle were completed using the same methods, which are shown in Figures 4 and 5.

The static analysis described above gave the input torques *T*_*a*_, *T*_*b*_, *T*_*c*_, *T*_*d*_, *T*_*P*_, and *T*_31_ that were needed to drive the bones when the shoulder was in a specific position and posture was under a certain external load, and all of the input torques were generated by shoulder muscles that are connected to the bones.

#### 2.2.2. Static Analysis of the Muscles

The input torque was produced entirely by the muscle bundles attached to the bones, so nine equations were established based on the torque balance conditions of all 31 muscle bundles.

The literature shows that only when the real muscle-fibre length is more than 1.5 times the optimal muscle-fibre length *L*_*o*_^*M*^ will the muscle lose its active function of contraction and begin to generate passive force [33]. In this paper, the muscles were in the normal stretching range and in a state of active contraction, so the muscle force was always a tensile force. Since the muscle paths were determined already in the musculoskeletal model using the obstacle-set method, the muscle line of action could be determined as well. Therefore, the direction of the muscle force acting on a bone is always from the feature point (origin point or insert point) to the nearest via point.

**Figure 6:**
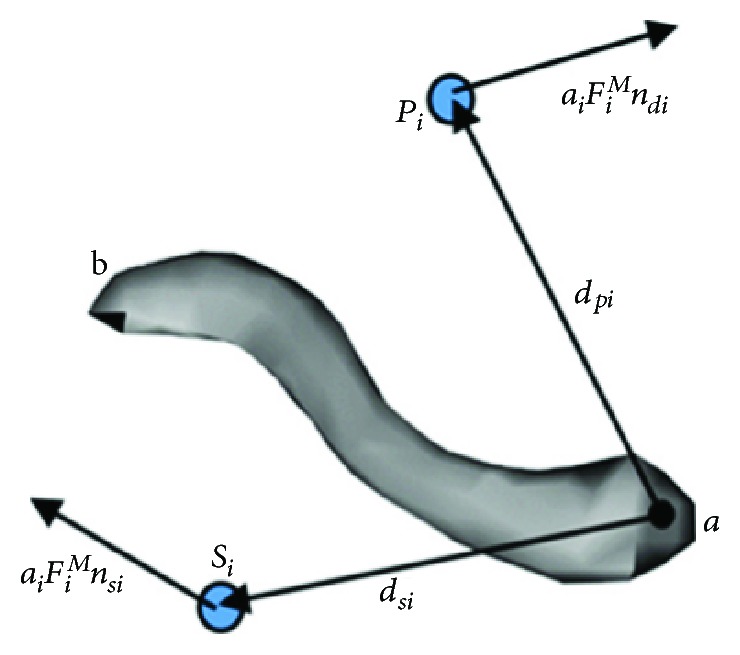
Free body diagram of the clavicle that considers muscle forces.

Figure 6 shows the condition of the muscle forces on the clavicle. The muscle bundles connected to the clavicle are M1, M5, M14, and M20; among these, M14 and M20 are connected to the clavicle at the origin point, while M1 and M5 are connected to the clavicle at the insertion point. In Figure 6, *a*_*i*_ is the activation of the muscle bundle Mi; *F*_*i*_^M^ is the isometric muscle force of Mi; *d*_*pi*_ is the vector from point *a* to the origin point of Mi, *d*_*si*_ is the vector from point *a* to the insert point of Mi; *n*_*pi*_ is the muscle force vector when Mi is connected to the bone by the origin point, *n*_*si*_ is the muscle force vector when Mi is connected to the bone by the insert point, and *n*_*pi*_ and *n*_*si*_ can be determined using the musculoskeletal model. Therefore, the actual muscle force of Mi can be represented as *a*_*i*_*F*_*i*_^*M*^*n*_*pi*_ when the feature point of Mi was the origin point (M14, M20) or *a*_*i*_*F*_*i*_^*M*^*n*_*si*_ when the feature point of Mi was the insertion point (M1, M5). The input torque *T*_31_ of the clavicle, which had already been calculated by the static analysis of the bones, was entirely produced by the muscle bundles above, so the torque balance equation could be given as follows:

(4) $$\begin{array}{c} \underset{i=14,20}{\sum }a_{i}{F_{i}}^{M}d_{pi}\times n_{pi}+\underset{i=1,5}{\sum }a_{i}{F_{i}}^{M}d_{si}\times n_{si}=T_{31}. \end{array}$$

**Figure 7:**
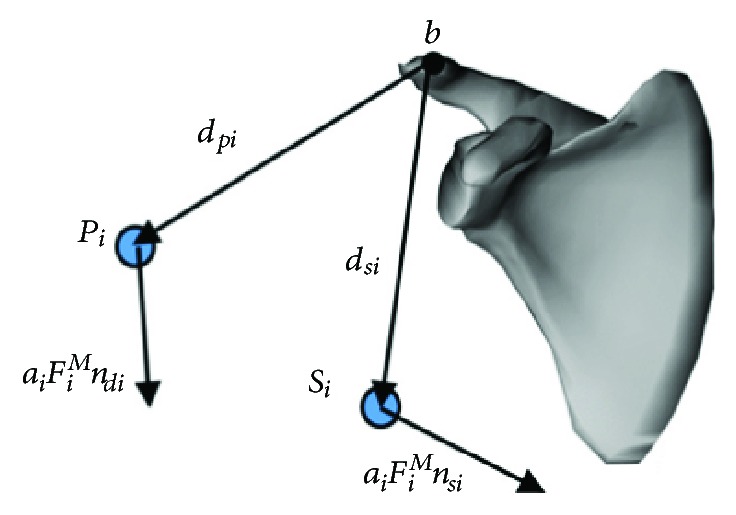
Free body diagram of the scapula that considers muscle forces.

The muscle bundles connected to the scapula could be analysed through the same process, as shown in Figure 7. The muscle bundles connected to the origin point were M21–M31, while the muscle bundles connected to the insertion point were M2, M3, M4, and M6–M13. The input torque *T*_*p*_ was entirely produced by these muscle bundles, so the torque balance equation could be given as follows:

(5) $$\begin{array}{c} \overset{31}{\underset{i=21}{\sum }}a_{i}{F_{i}}^{M}d_{pi}\times n_{pi}+\overset{4}{\underset{i=2}{\sum }}a_{i}{F_{i}}^{M}d_{si}\times n_{si}+\overset{13}{\underset{i=6}{\sum }}a_{i}{F_{i}}^{M}d_{si}\times n_{si}=T_{P}. \end{array}$$

**Figure 8:**
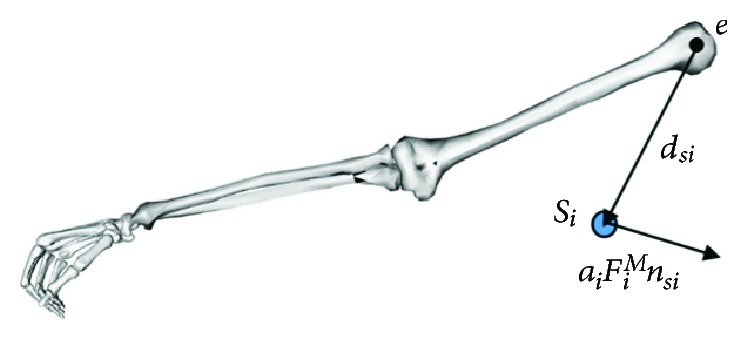
Free body diagram of the humerus that considers muscle forces.

The muscle bundles M14–M31, which are connected to the humerus, were analysed, as shown in Figure 8, where *S*_*i*_ was the adjacent feature point connected to the humerus. All of the feature points of the humerus were at the insertion point because the humerus is located at the end of the shoulder bones. The input torque *T*_*d*_ was entirely produced by these muscle bundles so the torque balance equation of the muscles could be given as follows:

(6) $$\begin{array}{c} \overset{31}{\underset{i=14}{\sum }}a_{i}{F_{i}}^{M}d_{si}\times n_{si}=T_{d}. \end{array}$$

By solving (4)–(6) simultaneously, nine equations were established to calculate muscle activation *a*_*i*_ (*i* = 1, 2,…, 31) in all 31 muscle bundles, so these problems had multiple solutions. According to the studies of Crowninshield and Brand [36], minimizing the sum of the squares of all muscle stresses was chosen as the objective function, so the multisolution problem above was transformed into an optimization problem with certain boundary constraints. The procedure of the static optimization was implemented using the FMINCON function in Matlab, and the optimal objective function was given as follows, where PSCA_*i*_ is the physiological cross-sectional area of the muscle bundle Mi given in Table 1:

(7) $$\begin{array}{c} FUN=\overset{31}{\underset{i=1}{\sum }}{\left(\frac{a_{i}{F_{i}}^{M}}{{PSCA}_{i}}\right)}^{2}. \end{array}$$

The real-time physiological cross-sectional area of a muscle bundle always changes during its contraction process, while the muscle belly volume (Vol) can be considered to be constant. A more appropriate optimal objective function, therefore, could be given as follows, where *L*_*i*_^*M*^ represents the actual muscle-fibre length:

(8) $$\begin{array}{c} FUN=\overset{31}{\underset{i=1}{\sum }}{\left(\frac{a_{i}{F_{i}}^{M}L_{i}^{M}}{{Vol}_{i}}\right)}^{2}. \end{array}$$

### 2.3. Algorithm of Optimal Load Orientation (OLO)

#### 2.3.1. Definition of the Optimal External-Load Orientation

**Figure 9:**
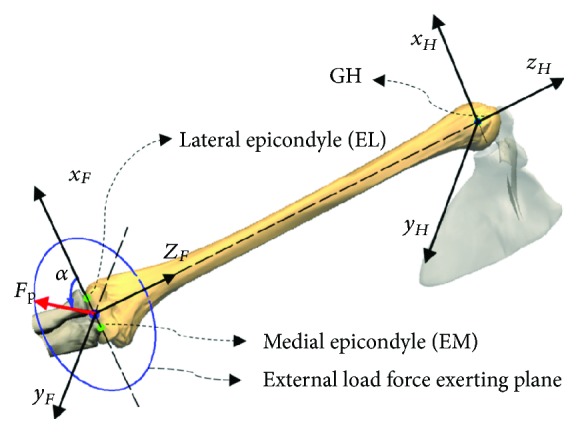
Schematic representing an external load applied to the shoulder.

The diagram of the upper limb under an external load *F*_*P*_ is shown in Figure 9. The force coordinate system *x*_*F*_*y*_*F*_*z*_*F*_, the origin of which was located at the midpoint of the EL and EM, was parallel to the humerus' frame of reference *x*_*H*_*y*_*H*_*z*_*H*_, which was defined in [25]. The upper arm and forearm were assumed to be relatively static, and the external load that was considered to be a pure force was assumed to be applied on the origin of the coordinate system *x*_*F*_*y*_*F*_*z*_*F*_. During shoulder movement, only when the direction of the external load *F*_*P*_ was perpendicular to the long axis of the humerus would the shoulder muscle suffer the greatest load effect. Therefore, the external load, the orientation of which was described by the angle *α*, was assumed to be in the normal plane and was considered to be perpendicular to the long axis of the humerus.

If a shoulder was moving under an external load with a constant magnitude, the muscle forces would depend on the posture of the shoulder and the orientation of the external load. Therefore, for a specific muscle to be active in a certain posture of the shoulder under a constant-sized external load, there would always be a specific external-load orientation that would lead to maximal muscle activation. This orientation of the external load was defined as the optimal external-load orientation (*α*_opt_).

**Figure 10:**
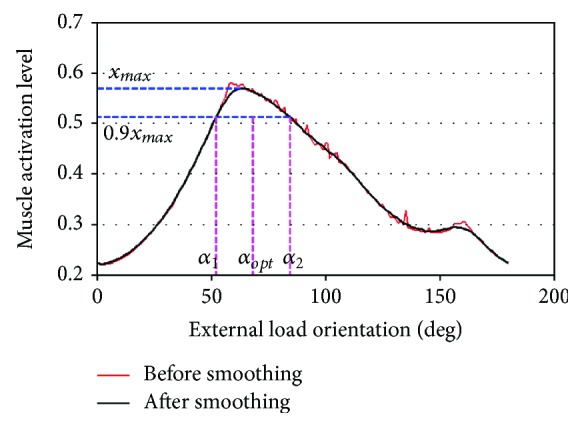
*x* − *α* curve of the deltoid acromial (DLTa).

The *x* − *α* curve shows the relationship between the level of muscle activation and the orientation of an external load. For example, the curve of a deltoid acromial activation (DLTa) with an external-load magnitude of 2.0 kg (approximately 20 N) is depicted in Figure 10 (where an abduction of 70 deg is performed). The activation plotted with a black line was the result of low-pass filtering (using a moving average filter with the span of 15). [*α*_1_, *α*_2_] was the load orientation interval that located the activation level in the interval range from 90% to 100% of its maximum. Thus, the midpoint of the interval [*α*_1_, *α*_2_] was defined to be the optimal load orientation of DLTa.

#### 2.3.2. Algorithm Expressing the Optimal External-Load Orientation

For a certain posture of the shoulder, this calculation results in *α*_opt_ that varies with the magnitudes of *F*_*P*_ in several cases. For some shoulder postures, when the assumed value of *F*_*P*_ was small, the *x* − *α* curves of some muscles would appear to be impulse noise around its maximum.Due to the small value of activation and narrow bandwidth of *α*_opt_, calculated activations less than 0.02 were considered unreliable and discarded. For instance, the activation level of the triceps brachii long (TRCl) head tended to be very low (<0.02), with an abduction from 20° to 40°, with an external load that was less than 2.5 kg (approximately 25 N). With the external load value increased to be greater than 2.5 kg, the activation of TRCl became significant and an optimal orientation cluster could be determined. Similar patterns happened with many other muscles, such as the supraspinatus, teres major, and deltoid scapular. Additionally, when the load value was assumed to be large, a well-distributed relatively high activation level might appear in the *x* − *α* curves, thus resulting in uncertainties relating to *α*_opt_. Different load magnitudes could result in different distributions of *α*_opt_. The analysis results showed that *α*_opt_ changed with external-load magnitude but did not change much in certain intervals of external-load magnitudes (0.5~6.0 kg in this paper), which means that the distribution of *α*_opt_ has significant consistency over a range of loads. To obtain more complete results, *α*_opt_ was calculated under different external loads (*F*_*P*_ = 0.5 kg, 1.0 kg, … , 6.0 kg), and the least squares-fitting result of *α*_opt_ values were calculated as the mean optimal external-load orientation (M − *α*_opt_).

**Figure 11:**
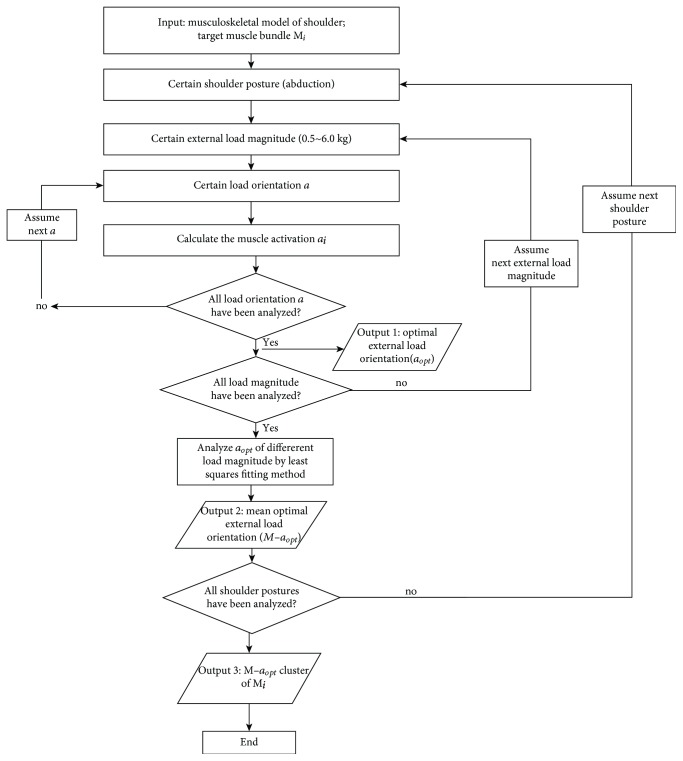
Flowchart demonstrating the optimal external-load orientation algorithm.

The flow chart shown in Figure 11 describes the algorithm used to determine the optimal external-load orientation. The *x* − *α* curve of a specific muscle under a certain magnitude external load for a certain shoulder posture was obtained through iteration, where the value of *α*_opt_ could be simultaneously determined. Another iteration was then performed by changing the load magnitude from 0.5 kg to 6.0 kg to calculate M − *α*_opt_. Finally, another iteration was completed to obtain all of the values of M − *α*_opt_ present in the whole trajectory of a rehabilitation movement (M − *α*_opt_ cluster) by changing the shoulder posture. The M − *α*_opt_ clusters of all of the muscle could be similarly determined.

#### 2.3.3. OLOC Training Simulation and Evaluation of the Training Trajectory

The weight-lifting abduction and abduction with OLOC under the same magnitude of external load were simulated to evaluate the effect of OLOC presented above to allow the promotion of the training efficiency. The mean activation over different abduction angles under a certain magnitude of external load was used to represent the training efficiency in a specific muscle.

For a given rehabilitation movement trajectory, the training efficiency differences among different muscles were evaluated, and the applicability of the rehabilitation movement for different muscles was also evaluated.

#### 2.3.4. Influence of the Load Magnitude

Different muscles' training effects are different in response to different load magnitudes. To determine the response characteristics of the external-load magnitude of the shoulder muscles, the mean activations of each main muscle bundle under different load magnitudes were calculated and compared.

To obtain the optimal load orientation cluster and study the influence of the load magnitude, shoulder abduction was simulated in the coronal plane from 20 to 80° with load magnitudes ranging from 0.5 kg to 6.0 kg, and the results were analysed.

## 3. Results

### 3.1. Optimal External-Load Orientation

**Figure 12:**
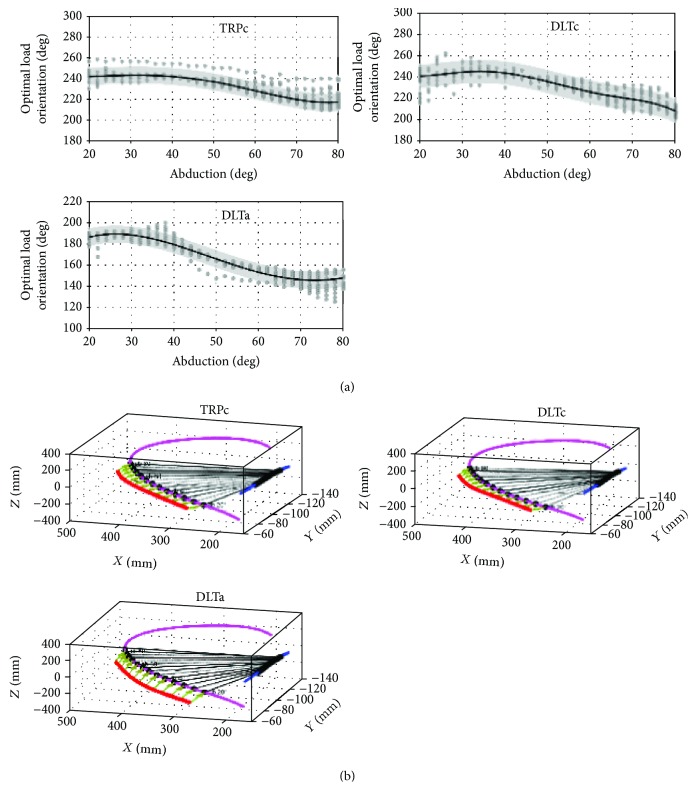
Optimal external-load orientation distribution of some muscles.

Despite different distributions of *α*_opt_ due to different load magnitudes for a specific muscle, the results indicated a significant consistency in the activations calculated under different magnitudes of external load.  Figure 12(a) shows the *α*_opt_ of TRPc, DLTc, and DLTa under a series of load magnitudes. The grey circle shows the distributions of *α*_opt_, and the solid line shows the M − *α*_opt_ curve obtained by regression fitting. The analysis suggested that within a certain range of loads, the distribution of *α*_opt_ in these muscle bundles showed good consistency and the values of M − *α*_opt_ were unique. The 3D representation of the M − *α*_opt_ results is shown in Figure 12(b), where the coordinate system used in this figure coincided with the ground reference described in [25]. The GH centre trajectory (blue line), elbow movement trajectory (pink line), and virtual long axis of the humerus (black dotted line) are all vividly shown. The red line and arrow represent the distribution of M − *α*_opt_. The distribution of M − *α*_opt_ has good continuity with the path of the elbow in space.

**Figure 13:**
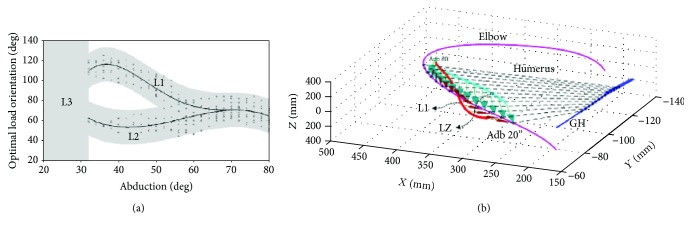
Optimal external-load orientation distribution of the teres major (TMJ).

Like the three muscle bundles described above, many other muscles' M − *α*_opt_ are distributed in a single region, such as PMJs, DLTs, and SUPR. However, except for scenarios involving a single region, there were also multiregion distributions for several muscles, including SRAm, TRPt1, LTDt, TMJ, and TRClg, among others.  Figure 13 shows the optimal load orientations of TMJ, where L1 and L2 were the two optimal orientation paths available for selection obtained by regression fitting. In this range of abduction, the muscle activation of TMJ caused by an external load with the same magnitude along L1 or L2 had no large difference (the difference is less than 10%). Moreover, the activations of TMJ performed uniformly during an abduction from 20 to 32 degrees (region L3).

### 3.2. Active-Resisted Abduction with Loads Based on OLOC

**Figure 14:**
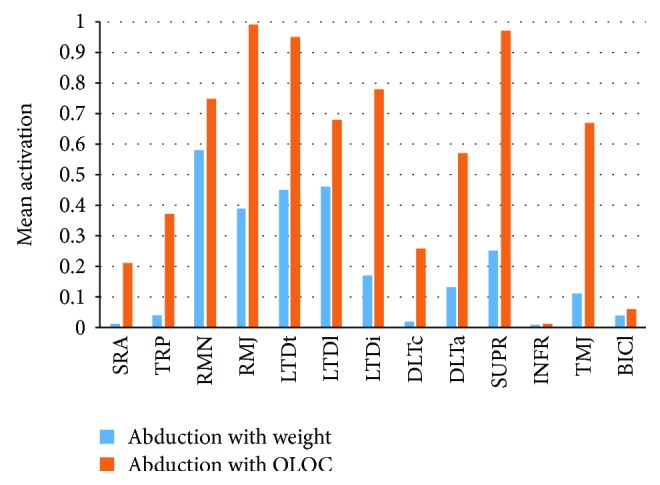
Comparison of the mean activations of the main muscles predicted by simulating weight-lifting abduction (blue) and abduction with optimal-orientation external load (orange).

Figure 14 shows a comparison of the mean activations for the main muscles of the shoulder obtained from a simulation of abduction with weight lifting of 3.0 kg that would always apply a vertical force on the humerus and abduction with OLOC under the same magnitude of load (*F*_*P*_ = 3.0kg). The results suggested that the muscle-specific rehabilitation training method based on the OLOC significantly promoted the training efficiency of specific muscles (the average of the increased proportion of the mean activation of all muscles was 537%, and the mean activation of all muscles was promoted by 165%).

There were several muscles whose mean activations were promoted more significantly, which included the SRA (from 0.00 to 0.21), LTDc (from 0.02 to 0.26, promote 1640% relatively), TRP (0.04 to 0.37, 905%), TMJ (0.11 to 0.67, 532%), LTDi (0.17 to 0.78, 361%), DLTa (0.13 to 0.57, 325%), and SUPR (0.25 to 0.97, 280%). There were some muscles whose promotion was only moderately increased but was kept at a relatively high level, including the RMN (75%), RMJ (99%), LTDt (95%), and LTDl (68%). However, there were also instances of an insignificant promotion of the mean activation, including the INFR (1.07%) and BICl (5.89%). The results of this comparison indicated that there were differences in the training efficiency among many muscles for the same rehabilitation movement using the OLOC.

### 3.3. Evaluation of Rehabilitation Training Movements

**Figure 15:**
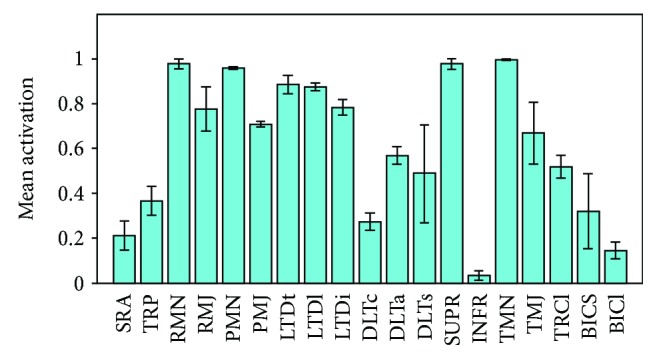
Average activations (mean ± *SD*) of the main muscles.

In an OLOC training movement of a certain trajectory, the mean activations of different muscles were different. The differences in activation were governed by the movement trajectories used in the shoulder's rehabilitation.  Figure 15 shows the average activation of different muscles in OLOC training (abduction from 20 to 80 degree, *F*_*P*_ = 3.0kg). The average activation is calculated by the mean activation and standard deviation (SD), which is shown as error bars.

On average, there were several muscles whose activations were relatively high (>0.8), which included the RMN (SD = 0.04), PMN (SD = 0.01), LTDt (SD = 0.08), LTDl (SD = 0.03), SUPR (SD = 0.05), and TMN (SD = 0.01). These muscles' levels of training were much higher than those of the others; thus, the results indicate that abduction as a rehabilitation movement was beneficial for the training of these muscles. However, the effects of an abduction in the coronal plane for the training of some muscles whose activations were lower than 0.22 were ineffective, including the SRA, INFR, and BICI, and of these, mostly the INFR was most noteworthy (*x* = 0.15). Furthermore, considering that a small external load is generally used in rehabilitation training, the SRA, INFR, and BICI may not be efficiently trained.

### 3.4. Influence of the Load Magnitude on the Effect of OLOC Training

**Figure 16:**
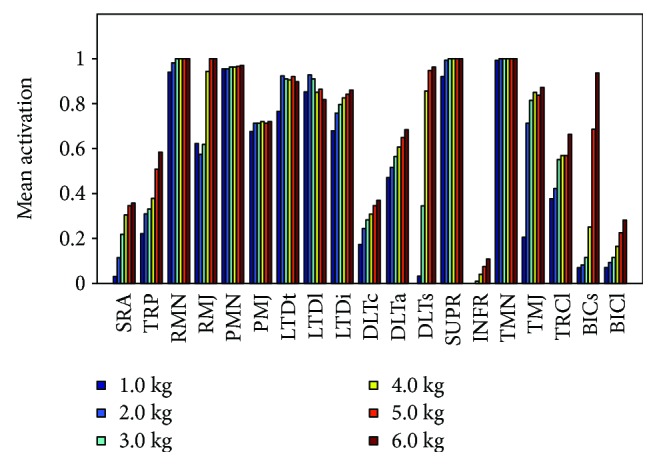
Effect of varying the external-load magnitude of the mean activations of the main shoulder muscles (bundles or groups) involved in abduction with OLOC.

**Figure 17:**
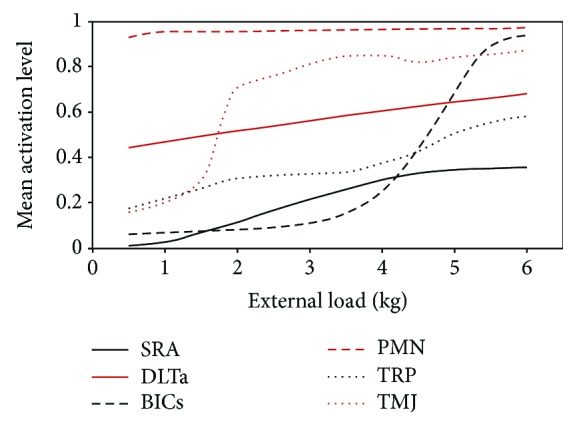
Impact of load magnitudes on the mean activations of some muscles.

For the aim of practical applications, an appropriate magnitude of external load must be determined before planning the rehabilitation.  Figure 16 shows the effect of varying the magnitude of the external load of the mean activations of the main shoulder muscles involved in abduction with OLOC, and Figure 17 depicts the impact of the load magnitudes on the mean activation for some muscles more intuitively.

There were some muscles whose activations showed a low level of activation when using a small load but experienced a significant promotion with an increase in load size, such as the SRA, DLTs, TRP, BICs, and other similar muscles. For these muscles, a small increase in the load could lead to a significant promotion in muscle activation. Some muscles' activations barely changed with the changing load magnitudes, such as the PMN, LTD, DLTc, and DLTa, among others. For these muscles, the training levels slowly promoted activity; thus, a change in the rehabilitation movement should be considered for these muscles. Therefore, when determining an external-load plan for training a specific muscle, an appropriate choice should be made between increasing the level of training and reducing the value of the load.

## 4. Discussion

The overall aim of this study was to propose a muscle-specific rehabilitation training method for the shoulder based on the optimal load orientation concept (OLOC). Biomedical engineering researchers developed human musculoskeletal movement models to reveal and imitate the structural characteristics and mechanisms that generate force in the musculoskeletal system. However, these results have not currently been fully utilized in the rehabilitation robots' field. Similarly, training strategies and training trajectories are often based on the clinical experience of a doctor or therapist rather than making precise decisions based on existing research results from a musculoskeletal model, so the training effects cannot be guaranteed and can be difficult to quantify and evaluate. Likewise, most of the rehabilitation robots designed to rehabilitate human limbs are designed to train an entire malfunctional limb rather than training specific muscles in it. Different muscles have different situations related to their damage, stiffness, or degeneration, so uniform training cannot produce the best training effects. There have been some studies on specific-muscle training [13–15], but the current research has great limitations because of the lack of use of the musculoskeletal movement mechanism. Therefore, a rehabilitation training method that is muscle-specific and based on the musculoskeletal model is proposed in this paper to provide a basis for the design of rehabilitation robots and further development of training strategies. Muscle activation was used in the musculoskeletal model to describe the force state on a specific muscle, and the optimal load orientation was proposed and calculated to maximize the training effect in some specific muscles.

The simulation results showed that training based on the OLOC could significantly improve the training effects in specific muscles more than simple weightlifting training. This method could be applied in rehabilitation robots designed to achieve a specific-muscle training function, which may significantly improve the practicality of using robots. This method also provided a method for quantifying the training effects on specific muscles during a given training process, which could be used to evaluate training effects and trajectory planning. For a specific muscle, an optimal rehabilitation movement may exist and can theoretically be designed to maximize the mean activation of the muscle.

Different muscles' training effects were different in response to different load magnitudes. The training effect on some muscles increased significantly with an increased magnitude of the external load, while the training effect in other muscles was much smaller. Therefore, when designing a rehabilitation training program for a specific muscle, a reasonable choice should be made between raising the training effect and reducing the external-load magnitude according to its characteristics of how the training effect responds to the external-load magnitude.

Bifurcation may occur to the spatial distribution of some muscles, which may provide more possibilities for designing rehabilitation robots and developing training strategies.

This paper focused on the analysis of shoulder muscles, but the same methods could be used in other joints of the human body as long as there is enough anatomical data.

## Figures and Tables

**Table 1:** Description of the reference frames of the shoulder.

Reference frame	Description
Global coordinate *S*_0_{*a* − *x*_0_*y*_0_*z*_0_}	The origin is located at point *a* on the SC articulation, and the axes *x*_0_,*y*_0_, and *z*_0_ are parallel to the coronal axis, sagittal axis, and vertical axis of the human body, respectively.
Clavicle coordinate *S*_1_{*a* − *x*_1_*y*_1_*z*_1_}	The origin is located at point *a* on the SC articulation. The *z*_1_ axis points to *b* from *a* along the clavicle axis, and the *x*_1_ axis is located horizontally and perpendicular to the *z*_1_ axis. The *y*_1_ axis can be determined by the right-hand rule.
Scapula coordinate *S*_2_{*b* − *x*_2_*y*_2_*z*_2_}	The origin is located at point *b* on the AC articulation. The *z*_2_ axis points to *c* from *b*, and the *x*_2_ axis is perpendicular to the *z*_2_*axis* and the scapular plane determined by *b*, *c*, and *d*. The *y*_2_ axis can be determined by the right-hand rule.
Thorax coordinate *S*_3_{*O* − *x*_3_*y*_3_*z*_3_}	The origin is located at point *O*, which is the centre of the thorax ellipse *S*_3_∣∣*S*_0_.
Humerus coordinate *S*_4_{*e* − *x*_4_*y*_4_*z*_4_}	The origin is located at point *e*, which is the centre of GH articulation. The *z*_4_ axis points to *e* from *f* along the humerus axis, and the *y*_4_ axis is perpendicular to the *z*_4_*axis* and the plane determined by *e*, *EL*, and *EM*. The *x*_4_ axis can be determined by the right-hand rule.

**Table 2:** Assumed musculotendon parameters of all 31 muscle bundles.

Number	Muscle bundles	Abbr.	Vol (cm^3^)	PCSA (cm^2^)	*L*_*o*_^*M*^ (cm)	*L*_*s*_^*T*^ (cm)	*F*_*o*_^*M*^ (N)	*β* (deg)
1	Subclavius	SBCL	8.80	4.36	2.02	5.07	144.02	0
2	Serratus anterior superior	SRAs	92.20	8.12	11.35	0.27	268.05	0
3	Serratus anterior middle	SRAm	71.71	4.00	17.91	0.75	132.12	0
4	Serratus anterior inferior	SRAi	194.65	8.41	23.15	0.01	277.51	0
5	Trapezius from cervical v1–6	TRPc	116.23	6.24	18.62	0.48	205.95	0
6	Trapezius cervical v7	TRPc7	77.49	3.61	21.44	0.60	119.25	0
7	Trapezius thoracic v1	TRPt1	66.92	3.45	19.37	0.32	114.01	0
8	Trapezius from thoracic v2–7	TRPt	197.25	12.40	15.91	0.42	409.23	0
9	Levator scapulae	LVS	71.92	3.78	19.02	0.90	124.78	0
10	Rhomboid minor	RMN	117.77	6.71	17.55	0.44	221.51	0
11	Rhomboid major thoracic v1–2	RMJt2	72.27	4.14	17.47	0.67	136.48	0
12	Rhomboid major thoracic v3–4	RMJt3	45.50	2.48	18.33	0.24	81.93	0
13	Pectoralis minor	PMN	73.14	4.87	15.03	0.01	160.55	0
14	Pectoralis major clavicular	PMJc	235.09	10.38	22.65	0.45	342.46	0
15	Pectoralis major sternal	PMJs	243.34	14.68	16.58	9.03	484.35	0
16	Pectoralis major ribs	PMJr	197.97	11.14	17.76	9.58	367.78	0
17	Latissimus dorsi thoracic	LTDt	183.23	5.26	34.87	14.75	173.43	0
18	Latissimus dorsi lumbar	LTDl	197.25	12.40	34.78	19.92	173.87	0
19	Latissimus dorsi iliac	LTDi	183.23	3.80	48.17	10.89	125.52	0
20	Deltoid clavicular	DLTc	123.48	8.41	14.69	1.64	277.48	0
21	Deltoid acromial	DLTa	376.94	56.38	6.69	8.56	1860.52	0
22	Deltoid scapular	DLTs	292.45	17.19	17.02	5.93	567.15	0
23	Supraspinatus	SUPR	89.23	20.84	8.3	3.1	354.77	0
24	Infraspinatus	INFR	225.36	33.32	6.76	5.58	1099.61	0
25	Subscapularis	SBSC	318.52	35.69	8.92	4.94	1177.93	0
26	Teres minor	TMN	38.70	6.77	5.72	4.55	223.35	0
27	Teres major	TMJ	231.40	15.59	14.84	5.79	514.51	0
28	Coracobrachialis	CRCB	80.01	4.55	17.60	4.23	150.05	0
29	Triceps brachii long head	TRClg	290.67	40.52	15.24	19.05	629.21	15
30	Biceps brachii short head	BICs	182.92	13.99	13.07	22.98	461.76	10
31	Biceps brachii long head	BICl	182.92	11.91	15.36	22.93	392.91	10

## Data Availability

Most of the parameters of the shoulder model could be found in the website https://simtk.org/projects/dsem.
